# T198. A SCHIZOPHRENIA-LIKE BIRTH SEASONALITY AMONG MATHEMATICIANS AND AN OPPOSITE SEASONALITY AMONG BIOLOGISTS: MORE EVIDENCE IMPLICATING BIMODAL RHYTHMS OF GENERAL BIRTHS

**DOI:** 10.1093/schbul/sby016.474

**Published:** 2018-04-01

**Authors:** Giovanni Marzullo

**Affiliations:** Per Aspera Research Foundation

## Abstract

**Background:**

Based on early-20th century births, a pre-electric illumination time of comparatively normal human exposure to sunlight, studies of schizophrenia (SCZ) found a birth seasonality with two opposite effects: a SCZ-liability peak among subjects born around late-February and an equally significant SCZ-resistance peak among those born six months later, around late-August. We previously investigated this rhythm in connection with a sunlight-dependent bimodal rhythm of general births that, prior to the full advent of electric lighting (but not later), occurred ubiquitously in non-equatorial parts of the world. We found that the SCZ-liability peak coincided with a first, Feb-Mar peak of general-population births (the GP1) while the SCZ-resistance peak coincided with a second, Aug-Sep peak of those births (the GP2). Moreover, in a study of hand and visual-field preferences among professional baseball players, we found the SCZ-liability, GP1-coincident seasonality among players with preferences denoting cerebral asymmetry “deficits” (CADs) and the SCZ-resistance, GP2-coincident seasonality among those with preferences denoting cerebral asymmetry “excesses.” Also, in a study suggested by associations of CADs with artistic abilities, we found the SCZ-liability, GP1-coincident seasonality among groups representing visual, performing and literary art “creators” (VPL-Artists) and the SCZ-resistance, GP2-coincident seasonality among groups representing art critics, historians, curators and other art “observers” (Para-Artists). Together, these findings suggested, as one possibility (but see later), that the SCZ-liability, CAD effects and artistic abilities could all three represent traits genetically or otherwise selected into the GP1 excess population of newborns and out of the GP2 population. The present study of “scientists” was initially aimed at the purported arts/science antithesis.

**Methods:**

Birth seasonalities were examined among early-20th century born American scientists and among yet earlier European biologists and mathematicians.

**Results:**

A group representing 1,925 American scientists showed the SCZ-resistance, GP2-coincident seasonality. However, this effect proved to be mostly due to biologists because biochemists, chemists, and physicists showed gradually less seasonality while mathematicians suggested an altogether artist-like, GP1-coincident seasonality. This intimation of a biologist-mathematician antithesis was pursued with an investigation of most major figures in the history of the two sciences from the 15th to the early-20th century. The two groups, numbering 576 mathematicians and 787 biologists, shared the same mean decade of birth, the 1780s, and essentially the same geographic origin in Western Europe. The mathematicians showed a very significant SCZ liability-like, GP1-coincident seasonality while the biologists showed an even more significant SCZ resistance-like, GP2-coincident seasonality. The latter effect was particularly strong among naturalists, anatomists and other groups representing biological “observationalism” as opposed to “experimentalism.”

**Discussion:**

The findings are discussed in light of a) new evidence that the annual photoperiod is indeed alone responsible for both peaks of general births, with the GP1 and the GP2 being caused by maternal periconceptional exposure to, respectively, the summer-solstice sunlight maximum and the winter-solstice minimum, and b) an approach/withdrawal theory of lateralization of basic emotions where the left cerebral cortex would handle external stimuli eliciting complacent emotions towards external realities while the right cortex would handle internal stimuli eliciting disdain for those realities.

